# Socio-demographic aspects and treatment-related factors on oral cancer patients’ participation in rehabilitation

**DOI:** 10.3205/iprs000168

**Published:** 2022-09-01

**Authors:** Philippe Korn, Simon Spalthoff, Joachim Hammersen, Gertrud Krüskemper, Frank Tavassol, Jan Winterboer, Fritjof Lentge, Nils-Claudius Gellrich, Philipp Jehn

**Affiliations:** 1 Department of Oral and Maxillofacial Surgery, Hannover Medical School, Hannover, Germany; 2 Department of Otorhinolaryngology, Head, Neck & Plastic Facial Surgery, Klinikum Bad Hersfeld, Germany; 3 Department of Medical Psychology, Ruhr University of Bochum, Germany

**Keywords:** oral cancer, rehabilitation, quality of life, socio-demographic aspects, surgical treatment, oncology

## Abstract

**Objectives:**

After resection of an oral carcinoma, patients are faced with physical, psychological, and socioeconomic challenges. Rehabilitation plays an essential role in patients’ reintegration into their social and professional environment. This study evaluated whether socioeconomic aspects affect oral cancer patients’ participation in rehabilitation treatment.

**Materials and methods:**

A retrospective analysis was conducted with 1,532 patients following surgical treatment of oral cancer during an international multicenter rehabilitation study of the German-Swiss-Austrian Cooperative Working Group on Maxillofacial Tumors using a questionnaire comprising disease-related and psychosocial items postoperatively and at least 6 months after surgery.

**Results:**

Only 35.4% of patients participated in rehabilitation. Age (p<0.001), sex (p<0.001), and marital status (p<0.05) significantly influenced participation in rehabilitation. Postoperative impairment (p<0.05) as well as quality of life (p<0.01) were significantly worse in patients who participated in rehabilitation. Nevertheless, this group of patients returned to work significantly more often, although later, than those who did not participate in rehabilitation (p<0.05).

**Conclusions:**

The findings show social inequalities and suggest a general undersupply of rehabilitative follow-up treatment in patients with oral cancer. More patients, especially older people and women should be referred to rehabilitation.

## Zusammenfassung

**Ziele:**

Nach Resektion eines Mundhöhlenkarzinoms stehen Patienten vor körperlichen, psychischen und sozioökonomischen Herausforderungen. Die Rehabilitation spielt eine wesentliche Rolle bei der Wiedereingliederung der Patienten in ihr soziales und berufliches Umfeld. In dieser Studie wurde untersucht, ob sozioökonomische Aspekte die Teilnahme von Mundkrebspatienten an einer Rehabilitationsbehandlung beeinflussen.

**Material und Methoden:**

Im Rahmen der internationalen multizentrischen Rehabilitationsstudie des Deutsch-Österreichisch-Schweizerischen Arbeitskreises für Tumoren des Kiefer-Gesichtsbereichs wurde mittels eines Fragebogens eine retrospektive Analyse von 1.532 Patienten mindestens 6 Monate nach operativer Behandlung eines Mundhöhlenkarzinoms durchgeführt.

**Ergebnisse:**

Nur 35,4% der Patienten nahmen an einer Rehabilitation teil. Alter (p<0,001), Geschlecht (p<0,001) und Familienstand (p<0,05) beeinflussten die Teilnahme an der Rehabilitation signifikant. Die postoperative Beeinträchtigung (p<0,05) sowie die Lebensqualität (p<0,01) waren bei Patienten, die an der Rehabilitation teilnahmen, signifikant schlechter. Dennoch kehrte diese Patientengruppe signifikant häufiger, wenn auch später, in den Beruf zurück als diejenigen, die nicht an der Rehabilitation teilnahmen (p<0,05).

**Schlussfolgerungen:**

Die Ergebnisse zeigen soziale Ungleichheiten und deuten auf eine generelle Unterversorgung bei der rehabilitativen Nachbehandlung von Patienten mit Mundhöhlenkarzinomen hin. Mehr Patienten, insbesondere ältere Menschen und Frauen, sollten zu Rehabilitationsmaßnahmen angeleitet werden.

## Introduction

Cancer of the oral cavity (oral cancer) is among the most common tumor entities, with a global incidence of 6.2 and 3.6 per 100,000 for men and women, respectively [1], [2], [3]. Over the past few decades, the survival rates of oral cancer patients have substantially improved [4]. Patients’ quality of life (QoL) has also decisively increased because of improvements in surgical treatment, aftercare, and rehabilitation [5], [6], [7], [8].

Radical resection remains the cornerstone of oral carcinoma therapy. Surgical therapy of the primary tumor includes resection and reconstruction of the defect, which frequently requires a microvascular anastomosed tissue transplantation [5]. These procedures often lead to aesthetic impairments and considerable functional restrictions (e.g., speaking or swallowing) that need to be addressed by various rehabilitative procedures [9]. Functional impairments may also occur during the surgical therapy of the draining lymph system in the course of neck dissection, for example, because of injuries to the accessory nerve, which result in deficits in shoulder-arm mobility [10], [11]. If necessary, surgery is followed by radiation or combined radiochemotherapy, which also burdens patients, for example, because of a lack of saliva [12], [13]. Thereafter, rehabilitation and tumor follow-ups take place for early detection of recurrence and assuring social integration [14], [15]. In addition to treatment of the physical issues via speech, language, and physical therapy, psychosocial support is an integral part of rehabilitation [16], [17].

Knowing the importance of the aforementioned postoperative measures, the aim of the present study is to examine the effects of treatment-related factors and socio-demographic aspects on patients’ participation in rehabilitation as well as the effect of rehabilitation therapies on patients’ clinical findings, QoL, and return to work. 

## Methods

### Design and participants

The data used in the study were obtained from the German-Swiss-Austrian Working Group on Maxillofacial Tumors (DÖSAK). Data from 1,532 patients were analyzed retrospectively as part of an international, multicenter study among 38 hospitals in German-speaking countries (the DÖSAK Rehab Study) [7], [8], [11], [18]. 

### Instruments

The Bochum patient questionnaire on rehabilitation was used, comprising 147 disease-related and psychosocial items. Patients were asked about their conditions at various times, as previously described in multiple publications [7], [11], [18]. Inclusion criteria were (1) being diagnosed with carcinoma of the oral cavity and (2) being treated by curative intended surgery at least 6 months before assessment regardless of adjuvant therapies, such as radiation or chemotherapy. 

In addition to epidemiological data, socioeconomic findings were also collected, such as participation in rehabilitation. A 5-point Likert scale was used to assess 15 items on the most common impairments following oral cancer treatment, including speech and swallowing disabilities or reduced mouth opening and shoulder mobility (0: *none*, 1: *minor*, 2: *moderate*, 3: *severe*, and 4: *very severe*). 

QoL was evaluated on a scale ranging from 0 (*very bad*) to 100 (*very good*). The time to return to work was also recorded and divided into five categories: after 3 months, after 6 months, after 12 months, over 12 months, and no return to work. In addition, the patients rated the rehabilitation process on a 3-point scale *(poor*, *moderate*, *good)*. 

### Statistical analysis

The statistical analysis of the data was carried out using SPSS for Windows (SPSS Inc., Chicago, IL, USA) and Sigmaplot (Systat Software Inc., San Jose, CA, USA). Mean values and standard deviations were determined for the descriptive statistics. Parametric tests were used to analyze both interval scales and ordinal scales. A t-test was used for independent samples. The p-values given are based on the χ^2^-test and ANOVA; a value of <0.05 based on a confidence interval of 95% was regarded as statistically significant. 

All procedures performed in the study were in accordance with the ethical standards of the Institutional Review Board at Ruhr-Universität Bochum, and with the 1964 Helsinki Declaration and its later amendments. All participants in the DÖSAK Rehab study provided informed consent and agreed to scientific use of their data.

## Results

### Patient cohort

Participants’ average age was 59.4 years, with women (M=62.6) being slightly older than men (M=58.2). The sex distribution was almost 3:1, with men being affected more often. Regarding cancer stage, 31.7%, 40.2%, 11.9%, and 16.2% of participants were classified accordingly to pT1–4, respectively. No lymph node metastases (pN0) were found in 60.8% of the participants. Locoregional metastasis of the cervical lymph nodes was observed in 39.1% of the patients. Distant metastases were only found in 0.7% of the cases. 

Approximately half of the cases (45.7%) were treated by surgical resection only, while 35.8% and 18.5% of the cases were treated with adjuvant irradiation and chemotherapy, respectively. Moreover, 64.6% of the patients did not participate in any rehabilitation therapy. In 20.8% of the cases, only one rehabilitation procedure was carried out, while multiple rehabilitation procedures took place for 14.6% of the sample. 

### Socio-demographic aspects

Rehabilitative follow-up treatment was performed in 34.1% of the younger patients aged up to 39 years. This rate initially increases with increasing patient age (40 to 49: 42.1%; 50 to 59: 43.9%). A decrease with age was then seen, as fewer patients in the 60 to 69 and 70 to 79 age groups took advantage of rehabilitation therapies (28.9% and 21.4%, respectively). This rate rose to 31.6% in elderly patients aged 80 and over. These differences between the age groups were highly significant (p<0.001), as shown in Table 1 (Tab. 1). 

Men (37.9%) accepted rehabilitation offers significantly more often than women (27.8%; p<0.001). Almost two thirds of the patients (66.2%) were married, while 11.8% were widowed, 12.0% were divorced or separated, and 10.0% were single. Less than half (39.9%) of the unmarried tumor patients carried out at least one inpatient rehabilitation procedure; this percentage dropped to 35.2% in married patients, and was highest (41.8%) in divorced or separated tumor patients. Widowed patients were the least likely to take part in rehabilitation offers (only 26.7% did). These differences were statistically significant (p<0.05). However, graduation levels did not seem to be decisive in determining rehabilitation participation and the difference was not statistically significant (p=0.076; Table 1 (Tab. 1)). 

### Tumor and treatment-related factors

The percentage of patients who underwent inpatient follow-up treatment was lowest among patients with a pT4 tumor (31.1%), although the differences with the other stages (pT1: 36.0%, pT2: 36.2%, pT3: 36.6%) were not statistically significant. The number of operations performed correlated positively with participation in rehabilitation (p<0.001). The treatment method also influenced participation in rehabilitation. About 29.3% of patients who underwent surgery only participated in follow-up treatments. This rate was significantly higher in patients who underwent additional radiation therapy or combined radiochemotherapy (43.0% and 37.4%, respectively; p<0.001; Table 1 (Tab. 1)). 

### Rehabilitation

In half of the cases (48.7%), a combination of active (e.g., speech and swallowing therapy or occupational therapy) and passive (e.g., massages and lymphatic drainage) rehabilitation therapies was used, with additional psychotherapy in 18.4% of the cases. Remarkably, passive therapy was performed with only 17.5% of the patients. Moreover, 6.4% of the patients participated exclusively in active therapy and 1.1% in psychological therapy only. Combined with active or passive therapy, the proportion of psychological therapy increased slightly (5.3% and 2.2%, respectively).

### Impairment and quality of life

The intensity of all evaluated impairments was rated higher in patients who participated in rehabilitation than in those who did not participate. This held true at both recording times (immediately postoperatively and at least 6 months later). Table 2 (Tab. 2) summarizes the extent of postoperative impairments as mean values. All differences were statistically significant. This is also reflected in the QoL: patients who participated in rehabilitation reported significantly lower QoL than those who did not (p=0.01), as shown in Table 3 (Tab. 3).

### Economic aspects

Inpatient rehabilitation influenced patients’ ability to work. At the time of data collection, 30.9% of the patients that underwent follow-up treatment had returned to work, while only 25.1% of patients without inpatient follow-up treatment had done so (p<0.05). There was also a difference in the time taken by patients to resume work: while the frequency of readmission in patients who did not undergo rehabilitation continued to decrease, it initially rose in patients after rehabilitation, but decreased again after 12 months (p<0.001; Table 4 (Tab. 4)).

### Patients’ satisfaction with rehabilitation

The majority of patients (75.1%) rated the inpatient rehabilitation as good. This rate increased as the number of rehabilitation therapies increased. The differences were statistically significant among those who received one, two, or more rehabilitation therapies (p<0.001; Table 5 (Tab. 5)). 

## Discussion

Tumor patients in general, and patients after resection of an oral carcinoma in particular, are faced with physical, psychological, and socioeconomic challenges [19], [20]. Thorsen et al. have reported on the need for rehabilitation of over 60% of different tumor patients, with physiotherapy being the procedure most often needed [21]. However, in the present investigation, only 35% of patients were admitted to a rehabilitative follow-up treatment, which suggests an undersupply of the examined populations. Other authors have reported even lower rates in head and neck cancer [22]. Consistent with the needs expressed by other tumor patients, most of the patients used an active form of therapy [21].

### Socio-demographic aspects

Various studies have shown that women express the need for rehabilitation more frequently [19], [23]. Significantly, in our study, fewer women participated in rehabilitation therapies than men. Undersupply and sex-specific differences should be considered when considering rehabilitative measures.

Married patients were less likely to participate in rehabilitation. This was in line with other reports on the reduced need for rehabilitation in married patients [21]. It seems that the spouse’s help is more likely to be requested than that of a stranger and being in the home environment is preferred after a hospital stay, which may be more possible with spouse support. 

Age in general appeared to play a role in the use of rehabilitation measures: older patients tended to participate less frequently in rehabilitation, which is consistent with previous studies [24]. This may be due to a decreased interest in rehabilitation in older patients, which may also relate to widowed patients tending to be older and less likely to participate in rehabilitation [23].

Graduation also appears to influence participation in rehabilitation, which was explained, among other things, by an increased need and active search for information [23]. However, as a single factor, education level did not seem to have any effect in the present study.

### Tumor and treatment-related factors

Surprisingly, tumor size had no influence on rehabilitation participation. The assumption that an increased tumor size is accompanied by an increased degree of impairment, which in turn leads to an increased need and participation in rehabilitation could not be demonstrated in our study. It is also conceivable that socio-demographic influences mask the effect of tumor size.

The number of operations, however, correlated strongly with rehabilitation participation. An increased complication rate could have been decisive here, which could have led to more operations and consequently, an increased need for rehabilitation. It can also be assumed that multiple operations lead to greater functional impairments and psychological stress, which in turn results in increased use of rehabilitation.

It is also striking that previous radiation therapy led to more frequent rehabilitation, which might be due to the known radiogenic side effects, leading to an increased degree of impairment [25], [26]. Previous research found the lowest participation in rehabilitation in patients with radiation alone (not examined here) [22]. This was explained by the outpatient management of those patients and a decreased rate of admission to rehabilitation compared to inpatient treatment. Interestingly, additional chemotherapy played only a minor role in rehabilitation participation in our study, which is consistent with other studies [22].

### Impairment and QoL

Both immediately after the operation and at the time of data collection, patients who underwent rehabilitation therapies rated their impairments as more serious than those who did not. Despite the expected improvements after rehabilitation, the differences between the impairments of those who participated in rehabilitation and those who did not became bigger after follow-up treatment. This also affected the patients’ QoL, which was significantly worse, albeit to a lesser extent, in the group that participated in rehabilitation. An explanation for this was found in presumed negative selection, that is, it was not the rehabilitation itself, but rather a more severely impaired patient collective in this study’s rehabilitation group that may have caused these findings, as there is no doubt about the positive effect of rehabilitation on impairments and QoL in general [27]. 

### Economic aspects

As reported by other authors, the majority of patients did not return to work [28]. Participation in rehabilitation correlated with a significantly higher rate of returning to work. However, those who underwent rehabilitation tended to take more time to re-enter the occupational life than those who did not participate in rehabilitation. On the one hand, this delay can be explained in terms of the duration of the rehabilitation itself; on the other hand, it might be that an increased impairment of the patients in this group played a role. An age effect would also reinforce this finding: older patients have a lower probability of resuming work and participating in rehabilitation [28], [29].

### Patients’ satisfaction with rehabilitation

The vast majority of patients rated the rehabilitation as good. The low rates of rehabilitation participation in this study did not seem to be due to a negative assessment of the therapy itself. As satisfaction increases with the number of rehabilitative measures, this positive effect should be considered when planning treatment, although this may be compounded by the fact that convinced patients are more likely to participate in further therapies.

## Conclusion

Rehabilitation is the cornerstone in the reintegration of tumor patients into their social and professional environments. The findings obtained in this study regarding the use of rehabilitation show clear social inequalities, for example, regarding age and sex, and suggest a general undersupply. Especially from the perspective of participants’ positive evaluation of rehabilitation, more patients, particularly older people and women, should be referred to follow-up treatments.

## Notes

### Prior publication

Part of the data were published in advance in German as part of a doctoral thesis at the Ruhr University of Bochum, Germany [30].

### Acknowledgments

The authors would like to thank all the staff from the participating departments: Basel, Berlin (Steglitz), Berlin (Virchow), Bochum, Bonn, Dortmund, Duisburg, Düsseldorf, Erfurt, Erlangen, Essen, Frankfurt, Freiburg, Greifswald, Halle, Hannover, Heidelberg, Homburg-Saar, Innsbruck, Kiel, Cologne, Krefeld, Linz, Lübeck, Mainz, Marburg, Munich (LMU), Münster, Osnabrück, Recklinghausen, Regensburg, Rostock, Saarbrücken, Siegen, Suhl, Ulm, Würzburg, and Zürich, as well as the DÖSAK. We thank Editage (https://www.editage.com/) for English language editing.

### Competing interests

The authors declare that they have no competing interests.

## Figures and Tables

**Table 1:**
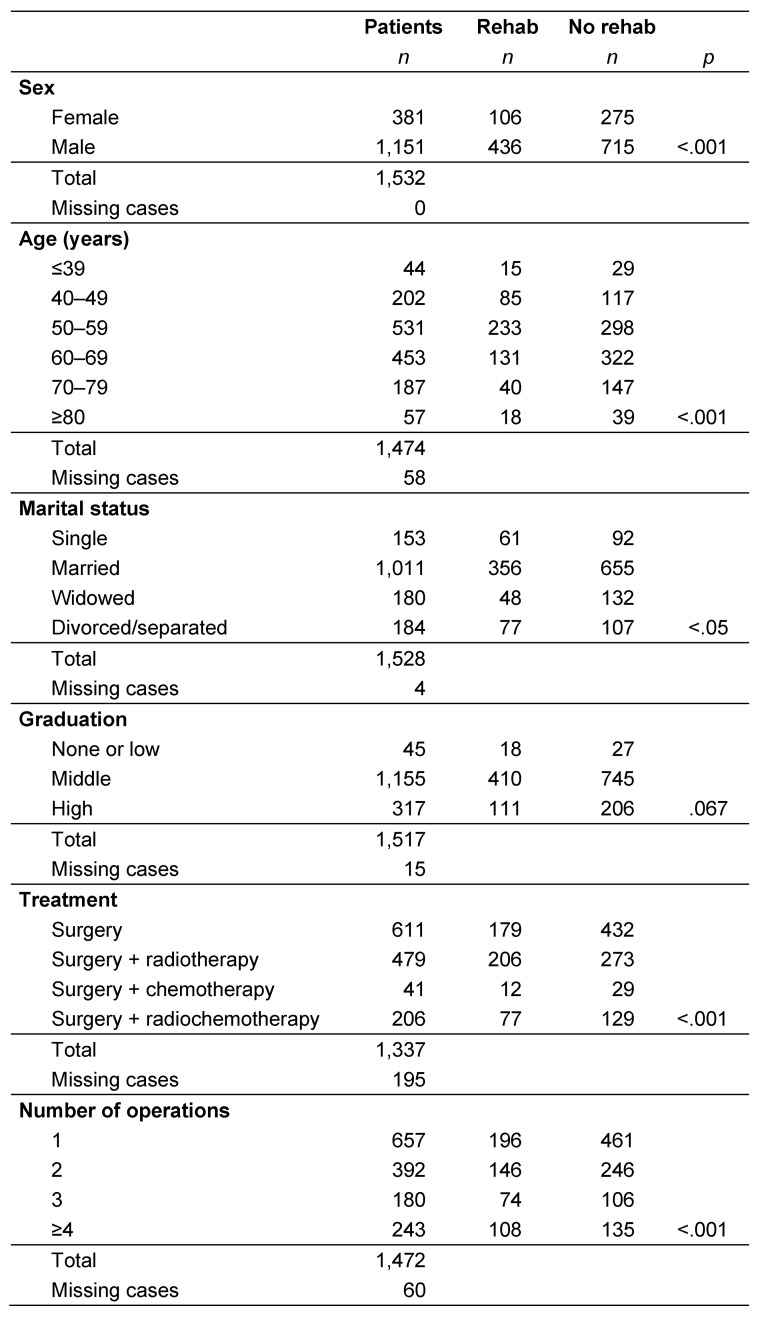
Correlation of participation in rehabilitation and treatment-related factors and socio-demographic aspects

**Table 2:**
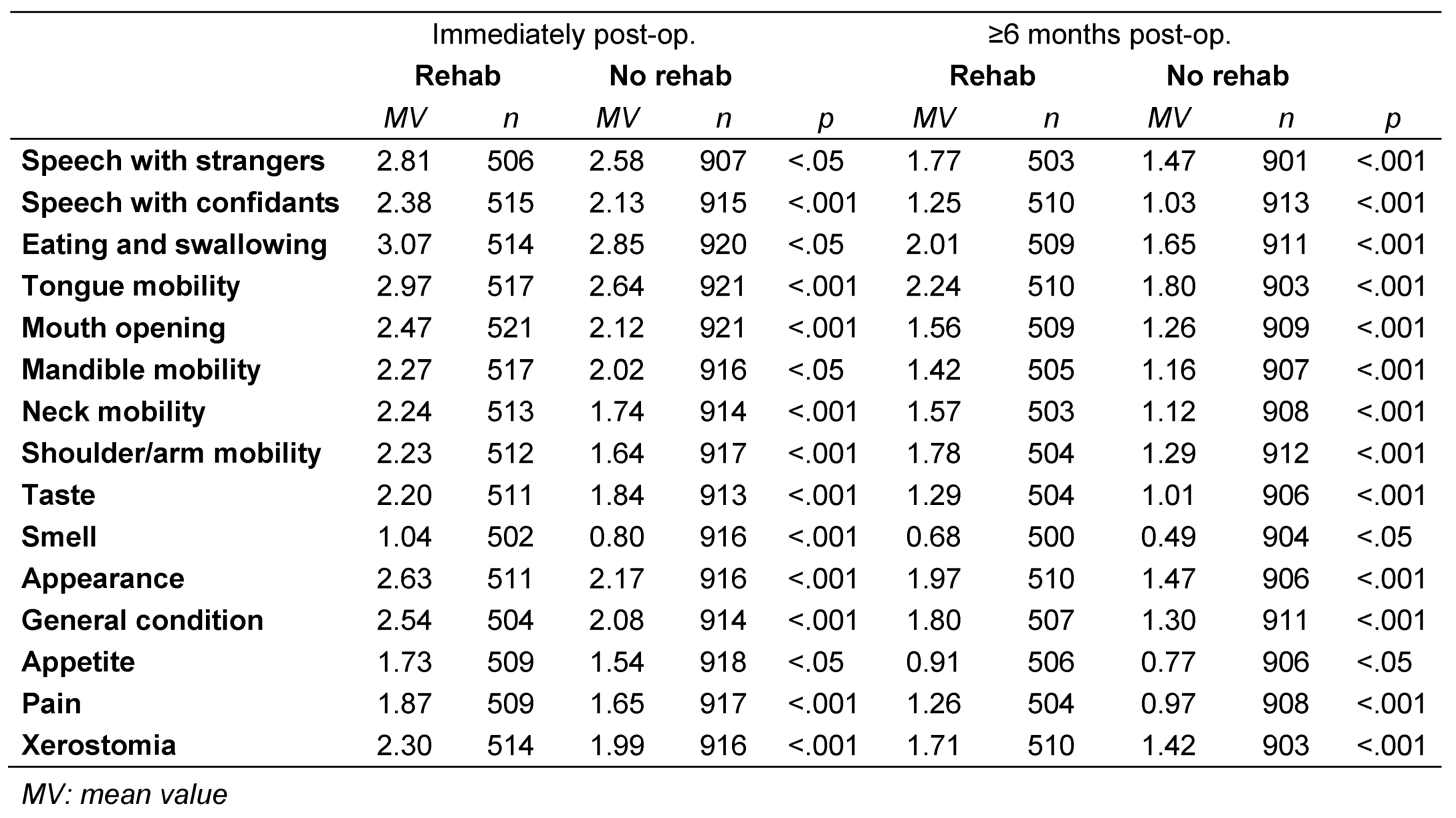
Severity of impairments and use of rehabilitation

**Table 3:**
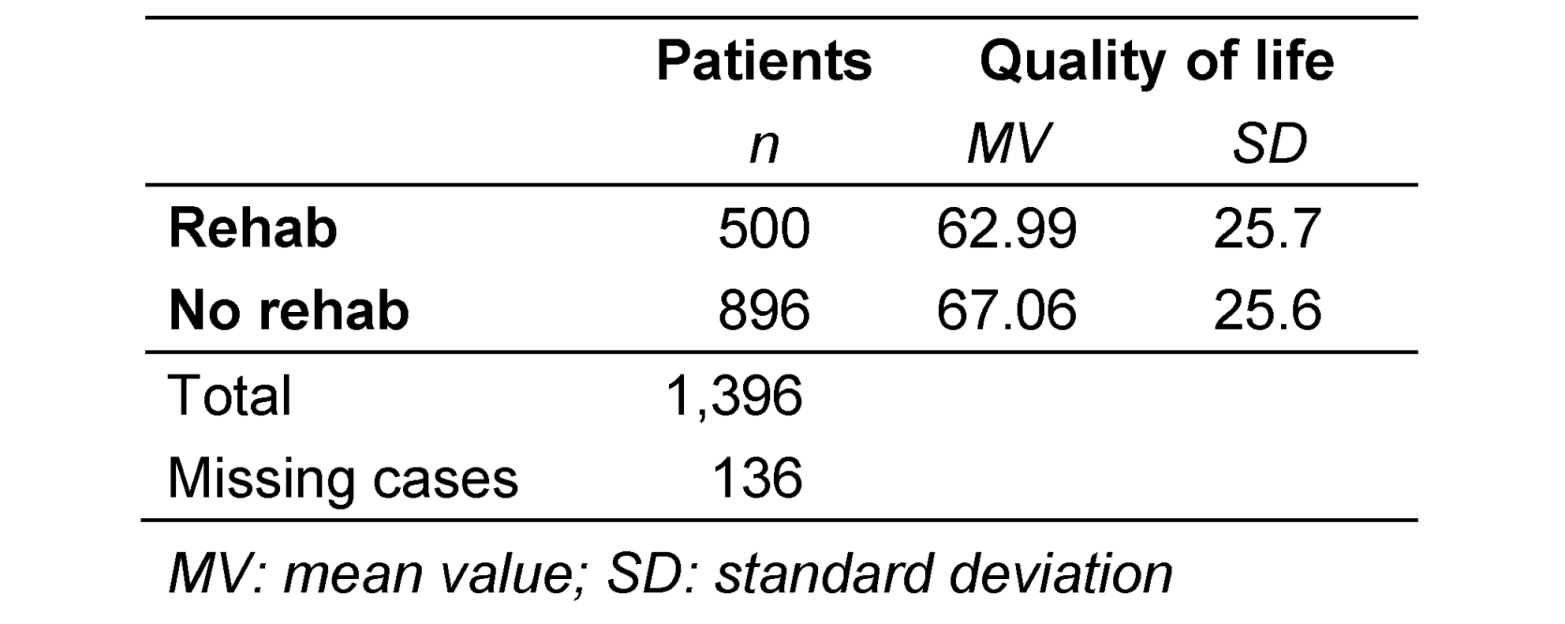
Quality of life and participation in rehabilitation

**Table 4:**
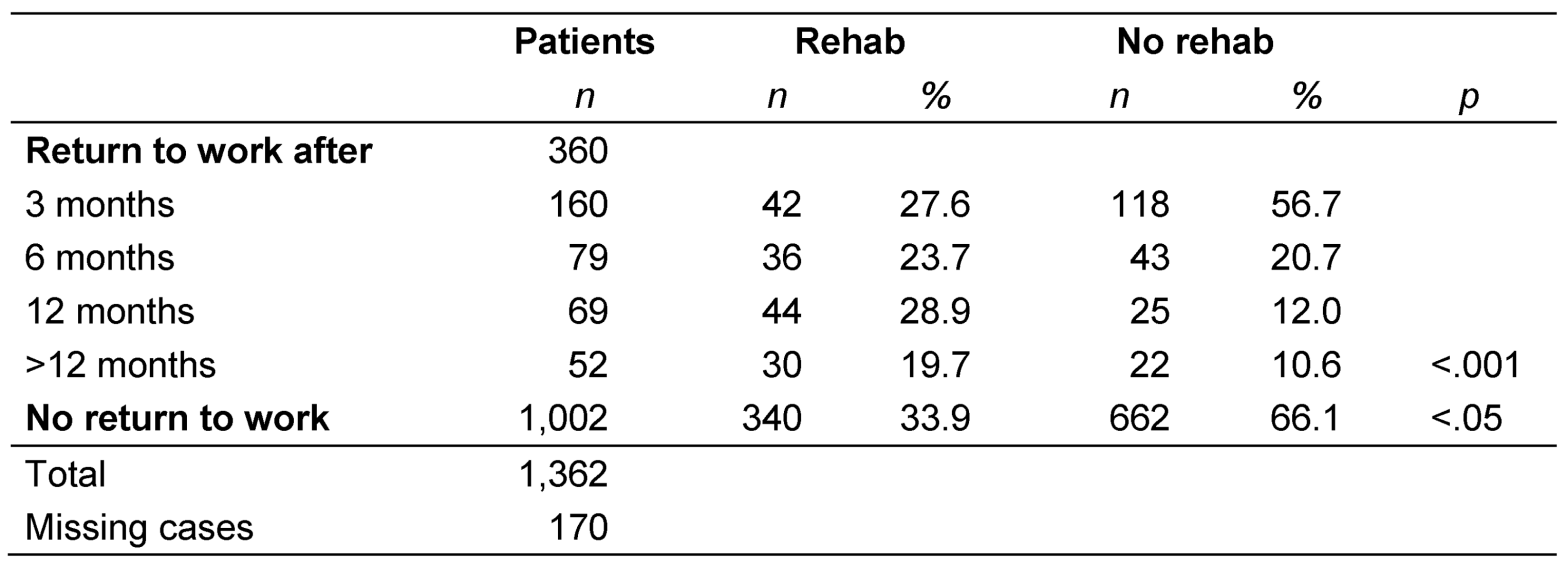
Time until return to work and participation in rehabilitation

**Table 5:**
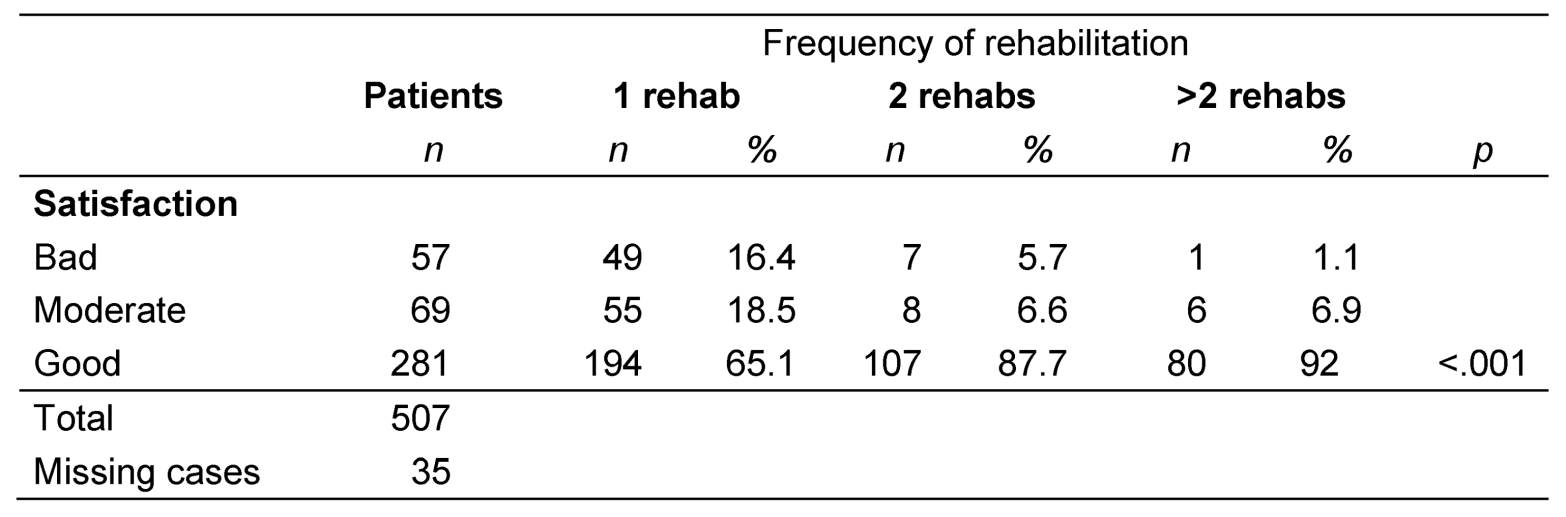
Patient satisfaction with rehabilitation
